# Haematotoxicity during peptide receptor radionuclide therapy: Baseline parameters differences and effect on patient’s therapy course

**DOI:** 10.1371/journal.pone.0260073

**Published:** 2021-11-18

**Authors:** Daphne M. V. de Vries–Huizing, Michelle W. J. Versleijen, Michiel Sinaasappel, Iris Walraven, Martine M. Geluk–Jonker, Margot E. T. Tesselaar, Jeroen J. M. A. Hendrikx, Berlinda J. de Wit–van der Veen, Marcel P. M. Stokkel

**Affiliations:** 1 Department of Nuclear Medicine, Netherlands Cancer Institute, Amsterdam, The Netherlands; 2 Department of Clinical Physics and Instrumentation, Netherlands Cancer Institute, Amsterdam, The Netherlands; 3 Department for Health Evidence, Radboudumc, Nijmegen, The Netherlands; 4 Department of Medical Oncology, Netherlands Cancer Institute, Amsterdam, The Netherlands; 5 Department of Pharmacy & Pharmacology, Netherlands Cancer Institute, Amsterdam, The Netherlands; IRCCS Ospedale Policlinico San Martino, Genova, Italy, ITALY

## Abstract

**Background:**

Mainly severe (CTCAE grade 3–4) haematotoxicity during peptide receptor radionuclide therapy (PRRT) is reported in literature due to major clinical impact, however moderate (CTCAE grade 2) haematotoxicity is common and could affect therapy management. The aim of this study was to evaluate the haematotoxicity course during PRRT and to compare baseline parameters between haematotoxicity grades.

**Methods:**

In this retrospective study, 100 patients with a neuroendocrine tumour treated with PRRT were included. Patients were treated with an aimed number of four cycles with 7.4 GBq [^177^Lu]Lu-DOTA-TATE administered every 10 weeks. Haematological assessment was performed at baseline and frequently up to 10 weeks after the fourth cycle. The lowest haematological value was graded according to CTCAE v5.0, and patients were classified using the highest observed grade. Differences in baseline parameters, including [^68^Ga]Ga-DOTA-TATE positive tumour volume, were evaluated between CTCAE grades.

**Results:**

Four cycles were completed by 86/100 of patients, 4/100 patients discontinued due to haematotoxicity, and 10/100 patients due to progressive disease. The treatment course was adjusted due to haematotoxicity in 24/100 patients, including postponed next cycle (n = 17), reduced administered activity (n = 13), and both adjustments (n = 10). The most observed haematotoxicity grade was grade 0–1 in 54/100 patients, grade 2 in 38/100 and grade 3–4 in 8/100. Significant differences in baseline leucocyte, neutrophil and platelet counts were observed between grade 0–1 and grade 2. However, the correlation between baseline and lowest observed values was poor to moderate. No differences between haematotoxicity grades and baseline parameters or somatostatin receptor positive tumour volume was observed.

**Conclusions:**

The incidence of severe haematotoxicity was low with extensive screening and monitoring. The vast majority of patients (96/100) was not restricted in treatment continuation by haematotoxicity; therefore, our selection criteria appeared appropriate for safe PRRT treatment. Baseline parameters showed limited correlation with the degree of decline in haematological values.

## Introduction

Peptide receptor radionuclide therapy (PRRT) for the treatment of neuroendocrine tumours (NET) was clinically established with the completion of the phase III NETTER-1 trial [1]. This trial showed increased progression free survival and improved quality of life in patients with a midgut NET treated with [^177^Lu]Lu-DOTA-TATE compared to patients treated with high dose octreotide only [2]. The red bone marrow and kidneys are the organs at risk during PRRT, as the peripherally circulating radiopharmaceutical irradiates the red bone marrow and is excreted via the urinary tract [1]. Long-term haematotoxicity after PRRT and persistent haematological dysfunction is described in 2.4–3.7% of patients with gastroenteropancreatic NET [1–7]. Monitoring of subacute haematotoxicity during PRRT is essential to maintain sufficient bone marrow function reserve [8], and significant relations between the grade of platelet toxicity during PRRT and the occurrence of myelodysplastic syndrome are observed [5].

The Common Terminology Criteria for Adverse Events (CTCAE) are used to evaluate the severity of adverse events [9]. In current literature, mainly severe (CTCAE grade 3–4) haematotoxicity during PRRT is described, as it has most impact on patient care. In PRRT, subacute grade 3–4 haematotoxicity is known for its association with impaired renal function, extensive tumour mass, high tumour uptake on pre-therapy imaging, age above 70 years and low baseline white blood cell counts [10]. Although literature on mild or moderate (grade 1 and 2) haematotoxicity is limited, it occurs frequently and affects PRRT treatments by required dosage adjustments and/or postponement of the next therapy cycle [1]. Hence, this could possibly lead to reduced therapy effect and accordingly worse survival.

Hypothetically, patients with extensive bone metastases have a higher risk for haematotoxicity due to high radioactivity accumulation in the bone. On the other hand, Beauregard *et al*. suggested that large tumour volumes would lead to decreased haematotoxicity, due to the so-called tumour sink-effect [11]. Tumour load segmented on pre-therapy [^68^Ga]Ga-DOTA-TATE PET/CT could be used to explore its role in subacute haematotoxicity during [^177^Lu]Lu-DOTA-TATE therapy. However currently there is no consensus concerning the optimal method for [^68^Ga]Ga-DOTA-TATE tumour volume delineation. A novel method for liver tumour volume segmentation was developed in our institute for [^18^F]FDG PET/CT, which could be applied for [^68^Ga]Ga-DOTA-TATE. Also, the current inclusion criteria for PRRT are strict with high sufficient bone marrow reserve.

The first aim of this study was to evaluate the haematotoxicity course during PRRT, including CTCAE grade 2 and grade 3–4 haematotoxicity, and the practical consequences for patient’s management. The second aim was to assess the correlation between baseline parameters and different haematotoxicity grades, with focus on receptor imaging derived tumour volume. Finally, our selection criteria with respect to haematological parameter values were evaluated for safe PRRT treatment.

## Materials and methods

### Eligibility for PRRT

The following inclusion criteria were used to evaluate eligibility for clinical treatment with PRRT: I) histopathological proven metastasized or irresectable well differentiated NET, II) increased somatostatin receptor expression, visualised on [^68^Ga]Ga-HA-DOTA-TATE (further noticed as [^68^Ga]Ga—DOTA-TATE), III) sufficient bone marrow function before and during PRRT, defined using the following thresholds: haemoglobin >5.5 mmol/L, leucocyte counts >3.0×10^9^/L, neutrophil granulocyte counts >1.0×10^9^/L and platelet counts >75×10^9^/L, and IV) kidney function measured by the glomerular filtration rate (GFR) above 50 ml/min/1.7m^2^, bilirubin maximal three times normal limit and serum albumin >30 g/L. The mentioned levels were based on the joint guideline by the EANM, SNMMI and IAEA and experience from the Rotterdam group [8,12]. Patients should not have been treated with previous PRRT cycles for inclusion in this study. The local Institutional Review Board approved this study (METC18.0684).

### [^68^Ga]Ga-DOTA-TATE PET/CT imaging and PRRT

[^68^Ga]Ga-DOTA-TATE PET/CT was performed within six months prior to the first PRRT cycle, ~45 minutes after an intravenous injection of ~100 MBq of in-house labelled [^68^Ga]Ga-DOTA-TATE [13]. PET images were acquired from mid-skull to mid-thighs and a low-dose CT was performed for attenuation correction and anatomical correlation. [^177^Lu]Lu-HA-DOTA-TATE (further noticed as [^177^Lu]Lu-DOTA-TATE) was labelled in-house according to validated protocols and in line with current Good RadioPharmacy Practice (cGRPP) guidelines [14]. Patients received up to four cycles of PRRT (7.4 GBq of [^177^Lu]Lu-DOTA-TATE per cycle) every 10 weeks, or reduced dosages and/or longer intervals between cycles in case of decreased haematological parameters. Long-acting somatostatin analogues (SSA) had to be discontinued at least four weeks before treatment and short-acting SSA for at least 24 hours before PRRT administration until 24 hours thereafter. An amino acid solution was infused during four hours for kidney protection, in concordance with ENETS guidelines [15].

### Image segmentation

Bone and soft tissue lesions were lesion-wise segmented using 40% of the standardized uptake peak value SUV_peak_, the 1 ml volume with the highest average uptake within a lesion [16]. Accordingly, the [^68^Ga]Ga-DOTA-TATE positive tumour volume in the bone and in soft tissue was derived. The [^68^Ga]Ga-DOTA-TATE positive liver tumour volume was determined using a novel method, which is described in detail in S1 Appendix. Briefly, all SUV-values in the liver were sorted in a histogram on which three Gaussian functions were fitted, presumed to represent the blood pool, normal liver parenchyma and tumour tissue. The normal liver parenchyma Gaussian fit was determined using visual assessment of the plots in combination with SUV measurements on [^68^Ga]Ga-DOTA-TATE PET/CT. All voxels values above the SUV average of the normal liver Gaussian plus one standard deviation were assumed tumour tissue. All segmentations were performed in 3D Slicer (version 4.10) and the liver histogram analysis was performed in using an in-house developed algorithm in Python.

### Data collection

The goal of the study was to evaluated subacute haematotoxicity, therefore, haemoglobin, leucocyte counts, neutrophil granulocyte counts and platelet counts were measured at baseline and at least every 3, 6 and 8.5 weeks after each treatment cycle until 10 weeks after the fourth PRRT cycle. Based on the laboratory assessment performed 8.5 weeks after each administration, was decided whether the next therapy cycle with 7.4 GBq [^177^Lu]Lu-DOTA-TATE was deemed safe or if adjustments were required. The lowest haematological value of each patient observed from the start of PRRT was graded to CTCAE v5. Patients were divided in CTCAE grade 0–1, grade 2, and grade 3–4 based on the highest observed grade in any of the four haematological parameters (haemoglobin, leucocyte counts, neutrophil counts, and platelet counts) at any time during PRRT [9]. The following baseline parameters were evaluated for association between CTCAE grades: gender, primary tumour site, tumour grade, [^68^Ga]Ga-DOTA-TATE positive tumour volume (liver, soft tissue, bone), co-morbidities, previous therapies, age at the time of the first PRRT administration, and baseline laboratory assessment. Diabetes and cardiovascular disease (hypertension, heart valve insufficiency, deep venous thrombosis, intermittent claudication and atrial fibrillation) were included as possible relevant co-morbidities due to known association with renal toxicity [3]. Laboratory values assessed in this study included haematological parameters (haemoglobin, leucocyte counts, neutrophil counts, and platelet counts) and baseline renal function (glomerular filtration rate, GFR).

### Statistical analysis

Parameters were evaluated for normal distribution using visual histogram assessment and non-normal distributed data was log transformed. Differences in baseline parameters between CTCAE grades were evaluated with Chi square, ANOVA and Kruskal-Wallis tests, with additional post hoc analysis in case of statistical significance (*p* values <0.05). Correlation and regression analysis were performed to assess the relation between baseline and the lowest haematological value during PRRT. Analyses were performed with SPSS statistics version 25 (IBM, Armonk, NY, United States) and Prism (GraphPad, San Diego, CA, United States).

## Results

This observational study included 100 patients treated with PRRT between March 2016 and March 2020. General patient characteristics are shown in Table 1. In our cohort, 50% of patients had grade 1–2 anaemia before the start of PRRT, of which 48/100 grade 1 and 2/100 grade 2. Baseline grade 1 leucocytopenia and thrombocytopenia was observed in 7/100 and 8/100, respectively. Four therapy cycles were completed by 86/100 of patients, while 9/100 and 5/100 of patients received three or two cycles, respectively. Of the patients who did not complete four therapy cycles, this was caused by persistent low haematological parameters in 4 patients: leucocyte counts of 2.9 and 2.2×10^9^/L in two patients, haemoglobin of 5.8 mmol/L in one patient, and in one patient leucocyte counts of 2.4×10^9^/L and neutrophil counts of 1.38×10^9^/L. In addition, 10 patients could not complete four cycles due to progressive disease during PRRT.

**Table 1:** General patient characteristics of the 100 included patients.

Characteristic		
Gender	Male	44
Age at start PRRT	Years (mean ± SD)	64 ± 10
Comorbidities	Diabetes	16
	Cardiovascular disease	59
WHO performance status	WHO 0	52
	WHO 1	39
	WHO 2/3	9
Primary tumour site	Ileum	55
	Pancreas	27
	Other*	18
Tumour grade	Grade 1	41
	Grade 2–3	59
Functional tumour	Number of patients	44
GFR	ml/min/1.7m^2^ (mean ± SD)	81 ± 20
CgA	μg/L [median, IQR]	846 [198–2320]
Bilirubin	μmol/L [median, IQR]	8 [5–10]
Haemoglobin	mmol/L [mean ± SD]	8.0 ± 0.9
Leucocyte counts	×10^9^/L [median, IQR]	6.8 [5.6–8.0]
Neutrophil counts	×10^9^/L [median, IQR]	4.2 [3.3–5.2]
Platelet counts	×10^9^/L [median, IQR]	244 [190–329]
Bone metastases	Number of patients	51
	volume (ml) [median, IQR]	11.8 [5.8–31.0]
Soft tissue metastases	Number of patients	96
	volume (ml) [median, IQR]	42.4 [19.2–96.6]
Liver metastases	Number of patients	91
	volume (ml) [median, IQR]	580.5 [318.1–1303.8]
Total tumour load	volume (ml) [median, IQR]	654.8 [388.6–1282.6]
Previous therapies	Primary tumour resection	43
	Loco-regional therapy	22
	Chemo- or targeted therapy	13
	Somatostatin analogues	80

IQR: Interquartile range.

*“Other” tumour sites were NETs of the lung, skin, rectum, caecum, retroperitoneal and unknown primary. Tumour volumes were derived from [^68^Ga]Ga-DOTA-TATE PET/CT imaging.

### Haematotoxicity during PRRT

**Fig 1:**
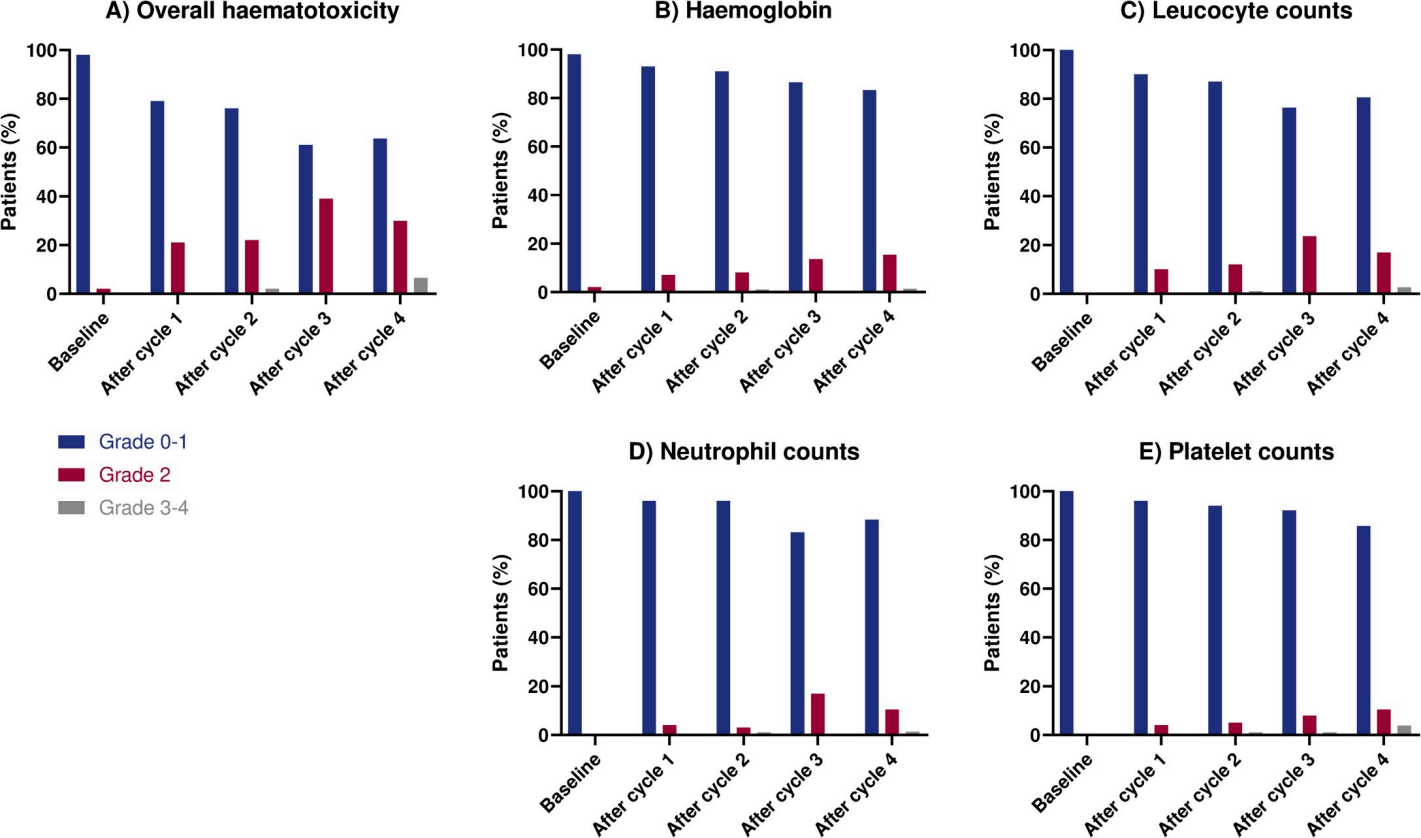
Haematotoxicity at baseline and after each treatment cycle according to CTCAE v5. A) shows overall haematotoxicity grades, indicating the highest toxicity grade observed in one or more of the haematological parameters which are further specified in (B-E).

Over all treatment cycles, any grade 2 haematotoxicity occurred in 38/100 patients, which was observed for the first time after the first therapy cycle in 18/38 patients, in 7/38 after cycle 2, in 10/38 after cycle 3 and in 3/38 after cycle 4. Grade 3–4 haematotoxicity was observed in 8/100 patients, of whom in 3/8 of patients after cycle 1, in 2/8 in cycle 2, 1/8 after cycle 3, and in 1/8 after cycle 4. The highest observed CTCAE grade per patient, haematological parameter and therapy cycle is shown in Fig 1, and the lowest observed value per haematological parameter and CTCAE grade during PRRT course in Table 2.

**Table 2:** The lowest observed value per haematological parameter and CTCAE grade during PRRT course.

	Grade 0–1		Grade 2		Grade 3–4	
	%	Median [IQR]	%	Median [IQR]	%	Median [IQR]
Haemoglobin	78	7.2 [6.6–7.8]	20	6.0 [5.7–6.1]	2*	4.3, 4.6
Leucocyte counts	74	3.8 [3.5–4.6]	23	2.5 [2.1–2.9]	3*	1.0, 1.9, 1.9
Neutrophil counts	80	2.5 [2.0–3.0]	18	1.3 [1.1–1.3]	2*	0.7, 0.9
Platelet counts	81	144 [111–219]	14	62 [57–73]	5	40 [29–49]

IQR = interquartile range. Haemoglobin in mmol/L, other parameters in ×10^9^/L. *small number of patients, therefore individual values separated.

**Fig 2:**
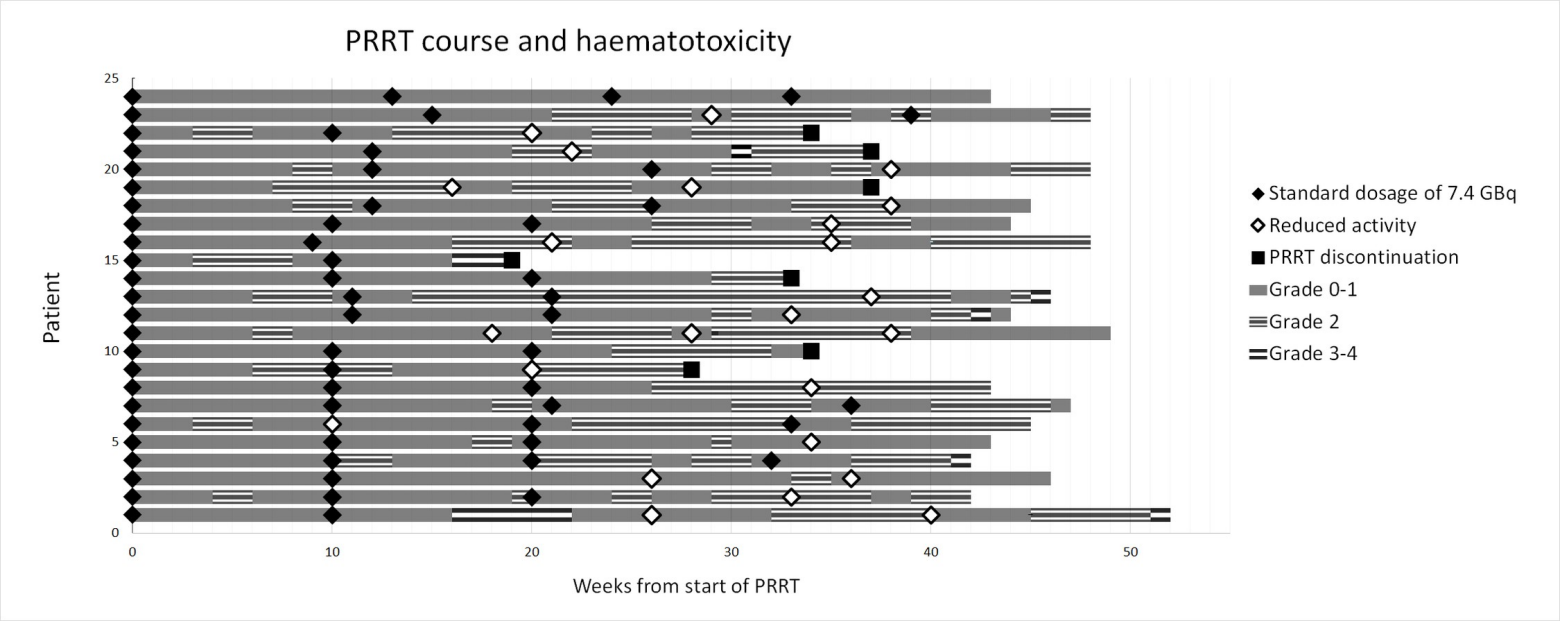
Swimmers plot of 24 patients with adjusted therapy schedules. The bar patterns represent the CTCAE grade and rhombus a normal or reduced dosage. In 17/24 (74%) patients the next cycle was postponed, in 13/24 (57%) patients the administered activity was reduced (5.5 or 3.7 GBq). In 10/24 (42%) patients both alterations were applied. In patient #3 the therapy was postponed and reduced activity was used due to fast declining platelets, without reaching grade 2 toxicity after the second cycle. In patient #21 the second cycle was postponed due to failure of [^177^Lu]Lu-DOTA-TATE production. Patient #9 discontinued PRRT due to acute renal failure and patient #19 and patient #21 due to progressive disease.

In 24/100 of patients the treatment schedule was adjusted due to haematotoxicity, of whom in 4 patients PRRT was permanently discontinued. The PRRT course and the development of haematotoxicity of these 24 patients are shown in the swimmers plot in Fig 2 and the occurred haematotoxicity is specified in S1 Table. In 17/24 patients the next cycle was postponed (range 12–18 weeks between cycles), in 13/24 patients the administered activity was reduced (range 3.7–5.5 GBq) and in 10/24 patients both adjustments were applied.

### Baseline parameter differences

Baseline leucocyte, neutrophil, and platelet counts values were significantly different between CTCAE groups, whilst baseline haemoglobin did not differ between the groups (Table 3). Other baseline patient and tumour characteristics, including the [^68^Ga]Ga-DOTA-TATE positive tumour volume, did not show any differences between the CTCAE groups (see S2 Table for specifications). Post-hoc analysis showed significant higher (*p* value of 0.004) baseline leukocyte counts in patients with any CTCAE grade 0–1 haematotoxicity than in patients with any CTCAE grade 2: 7.3 [IQR 6.3–8.3] vs. 5.8 [IQR 4.8–7.7]. The same pattern was observed for neutrophil granulocytes (*p* value of 0.002), patients with any CTCAE grade 0–1 had baseline neutrophil counts of 4.7 [IQR 3.8–5.4] and patients with any CTCAE grade 2 showed 3.7 [IQR 2.6–4.7]. The difference with respect to platelet counts was borderline significant (*p* value of 0.049) with 262 [IQR 215–342] being the baseline platelet counts for patient with any CTCAE grade 0–1 and 207 [IQR 170–327] for patients with any CTCAE grade 2 haematotoxicity. For all three haematological parameters can be observed that the baseline value for patients with grade 3–4 haematotoxicity was higher than in the group of patients with grade 2 haematotoxicity.

**Table 3:** Baseline values of haematological parameters corresponding to the highest observed CTCAE haematotoxicity grade of any hematologic parameter during PRRT.

Parameter	CTCAE grade			*p* value
	Grade 0–1	Grade 2	Grade 3–4	
Number	54	38	8	
Haemoglobin	8.1 ± 0.8	7.8 ± 0.9	7.6 ± 1.0	0.06
Leucocyte counts	7.3 [6.3–8.3]	5.8 [4.8–7.7]	6.9 [5.4–7.5]	0.006
Neutrophil counts	4.7 [3.8–5.4]	3.7 [2.6–4.7]	4.2 [3.4–4.8]	0.003
Platelet counts	262 [215–342]	207 [170–327]	218 [188–283]	0.048

Haemoglobin in mmol/L, other parameters in ×10^9^/L.

**Fig 3:**
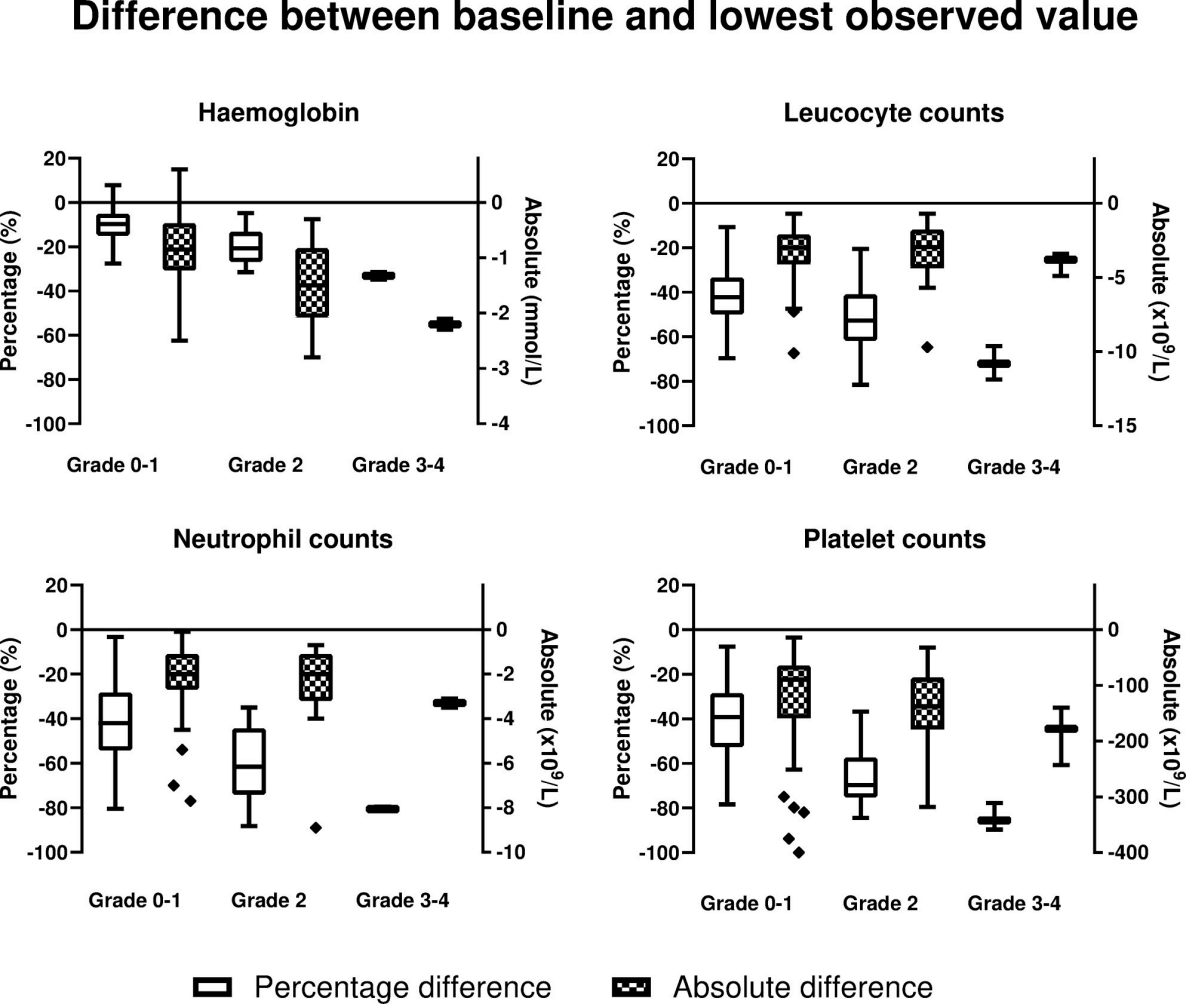
Percentage and absolute difference between baseline and lowest observed value per parameter and CTCAE grade. The horizontal line represents the median value.

**Fig 4:**
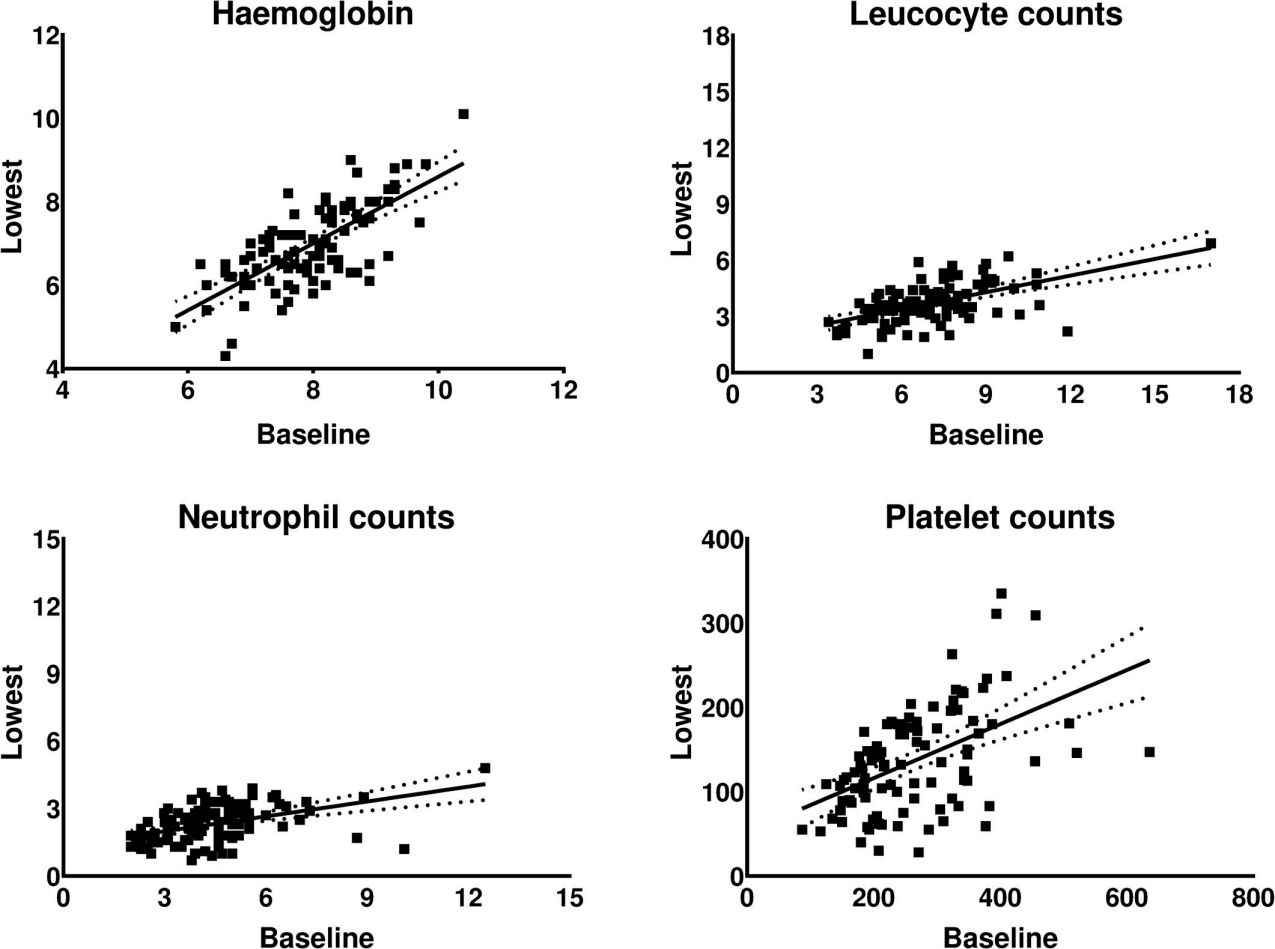
Scatter plots of baseline haematological values and the lowest observed value per patient, and the corresponding regression line. Haemoglobin in mmol/L, other parameters in ×10^9^/L.

The percentage and absolute difference between baseline and lowest observed haematological value is shown in Fig 3. This graph presents the largest absolute and percentage change between baseline and lowest value in patients with CTCAE grade 3–4 haematotoxicity. The baseline and lowest observed value per patient were significantly correlated (all *p* value < 0.001) in all four parameters. A moderate correlation of 0.679 and beta of 0.734 [95% CI 0.575–0.823] in the regression analysis was observed for haemoglobin, whereas the associations within the other parameters was lower, see Table 4. Scatter plots of the baseline and lowest observed value during PRRT are shown in Fig 4, along with the corresponding regression line.

**Table 4:** Correlations and regression analysis between the baseline and lowest observed haematological value.

	Correlation		Regression analysis		
	Coefficient	*p* value	Beta (95% CI)	R square	*p* value
Haemoglobin	0.679	< 0.001	0.734 (0.574–0.893)	0.461	< 0.0001
Leucocyte counts	0.536	< 0.001	0.294 (0.212–0.376)	0.334	< 0.0001
Neutrophil counts	0.491	< 0.001	0.223 (0.140–0.305)	0.227	< 0.0001
Platelet counts	0.528	< 0.001	0.322 (0.211–0.433)	0.252	< 0.0001

## Discussion

In this study, correlations between baseline parameters and subacute haematotoxicity during PRRT was assessed and the corresponding consequences for patient’s therapy course were evaluated. To monitor subacute haematotoxicity, laboratory assessments at baseline until 10 weeks after the fourth therapy cycle were included and long-term data was not collected. Prediction of subacute haematotoxicity during PRRT using baseline parameters is not possible based on our data. The low number of patients with severe haematotoxicity in this study could limit the power of the analysis. In our cohort, 95/100 patients could continue PRRT without consequences despite haematotoxicity with our current screening program and limits for haematological values. Still, close monitoring between treatment cycles is required and, if necessary, therapy adjustments should be applied. With 10-weeks intervals between consecutive [^177^Lu]Lu-DOTA-TATE administrations, a relative long recovery time is enabled. Although CTCAE grade 2 haematotoxicity is considered moderate with little clinical implications, it is a contraindication for treatment in some clinical trials [1,17]. A literature overview of studies reporting subacute haematotoxicity during PRRT is provided in Table 5. The differences in the observed CTCTAE grade 2 and grade 3–4 percentages between studies could be explained by differences in study population, since the inclusion criteria for clinical trials are usually stricter than for a routine clinical population. Mild baseline anaemia in the majority of patients was also observed by Medaer *et al*., as well as baseline grade 1–2 leucocytopenia in 7%, neutropenia in 6% and baseline grade 1 thrombocytopenia in 10% [18].

**Table 5:** Literature overview of subacute CTCAE haematotoxicity during PRRT (% of patients).

	Overall		Anaemia		Leucocytopenia		Neutropenia		Thrombocytopenia	
	G1-2	G3-4	G1-2	G3-4	G1-2	G3-4	G1-2	G3-4	G1-2	G3-4
Bergsma [10]		11		3.1		5.3				7.8
Del Prete [19]	96.2	63.5	21.2	7.7	55.8	5.8	26.9	3.8	48.1	5.8
Garske-Román [17]		15								
Löser [20]						3.3				3.3
Medaer [18]		45.1	83	18	52	7	51	6	46	14
Strosberg [1]			14	0	9	1	4	1	23	2
This study	88	8	83	2	64	3	18	2	60	5

In our cohort of 100 patients, the therapeutic schedules had to be adjusted due to haematotoxicity in 24 patients and in only four patients the treatment was permanently discontinued. In the study of Garske-Román et al., the white blood cell count should be >3.0×109/L and platelet counts >100×109/L. Based on these criteria, in 34.5% of patients one or more treatment cycles were postponed and PRRT was permanently discontinued in 22% of patients [21]. The platelet counts limits for continuing PRRT in our institute are less strict than in the study of Garske-Román, which could explain the smaller number of patients required adapted therapy courses due to haematotoxicity (21% vs. 34.5%). In the present study also the number of patients who had to discontinue therapy due to haematotoxicity was lower (3% vs. 22%). In the study of Garske-Román only delayed next therapy cycles were used, whereas in our study also reduced activities were administered.

Associations between subacute CTCAE grade 3–4 haematotoxicity and low creatinine clearance, age >70 years, extensive tumour mass, high tumour uptake compared to kidney uptake and baseline white blood cell counts <4.0×10^9^/L were observed by Bergsma *et al*. [10]. In our study, although statistically significant, a poor to moderate correlation between baseline haematological parameters and CTCAE haematotoxicity grade during PRRT was observed. The dispersion in the scatter plots of leucocyte, neutrophil and platelet counts was too large and resulted in lower R-square values than haemoglobin. Prediction of the degree of decline or the appearance of haematotoxicity during PRRT was not demonstration using baseline haematological values in our cohort. This could be caused by the low percentage of patients with grade 3–4 toxicity and 100 patients in this study.

We hypothesized that patients with high skeletal tumour load could have increased haematotoxicity due to increased irradiation of the bone marrow. On the other hand, a high overall tumour load could result in less haematotoxicity due to the ‘tumour sink effect’, since an inverse correlation between tumour dose and kidney dose was demonstrated in [^177^Lu]Lu-DOTA-TATE therapy [22]. In addition, an association between visual high tumour burden on Octreoscan and CTCAE grade 3–4 haematotoxicity was demonstrated [10]. Yet, the results in our study showed no relation between somatostatin receptor positive tumour volume and occurrence of subacute haematotoxicity during PRRT. The median tumour volume in the soft tissue and liver was higher in the CTCAE grade 3–4 group was than in the grade 2 group; however these volumes were higher in the grade 0–1 group than in the grade 2 group. The [^68^Ga]Ga-DOTA-TATE positive tumour volume in the bone was increasing from the grade 0–1 to grade 2 group, with the highest volume in the grade 3–4 group, however the differences were small.

Limited subacute severe (CTCAE grade 3–4) haematotoxicity is observed during PRRT, supporting the statement that PRRT is a safe therapy with the correct selection criteria [20,23]. Perhaps the administered dosage could be increased to deliver a higher dose to tumour lesions, on the other hand, fractionated treatments with lower amounts of administered activities with shorter time intervals could reduce bone marrow burden and have the same uptake in tumours lesions. For both approaches, close monitoring is required to ensure both safe and effective therapy, which does not alter from the traditional schemes and dosages. This monitoring could also include dosimetry, however the correlation between the absorbed dose to the bone marrow and decrease in haematological parameters is uncertain [17,24]. Simultaneously, kidney toxicity needs to be taken into account, although the effects are different per patient as kidney function declines per year and is more relevant in patients with additional risk factors [3]. Current expert’s opinion is that dosimetry should focus on bone marrow protection, since the kidney dose is limited and haematotoxicity could be especially relevant if more than four cycles are given [25]. However, Van der Zwan *et al*. reported similar incidence of CTCAE grade 3–4 haematotoxicity after 6 and 8 cycles of PRRT of 6.6% and 7.7%, respectively [26]. Another aspect that could be considered when aiming for reduction of normal tissue toxicity, is other therapeutic schemes. When the same absorbed dose is delivered to a certain tissue, the biological effective dose is lower if this dose is achieved in more fractions or administrations [27]. This common approach in radiotherapy enables normal tissue to recover when more fractions with lower amounts of radioactivity are given, while the radiosensitive tumour tissue still receives a good treatment [28].

Along with the retrospective nature of this study, the absence of correlation with dosimetry analysis is a limitation of this study. Ideally, the absorbed dose to the bone marrow is derived from blood samples, since this is currently the golden standard for bone marrow dosimetry. These analyses are labour intensive and therefore currently not included in our routine clinical workflow. Next to that, both our new segmentation method for [^68^Ga]Ga-DOTA-TATE positive liver lesions and the segmentation method for soft tissue and bone metastases could lead to an under- or overestimation of the true somatostatin receptor tumour volume. A consensus on this topic would improve the comparability between research projects, or the introduction of more artificial intelligence-driven techniques like whole-body tumour volume segmentations.

## Conclusion

PRRT in our hospital is safe therapy with low incidence of severe haematotoxicity using an extensive screening program and haematological monitoring, and 96/100 patients were not restricted in treatment continuation by haematotoxicity. No differences in baseline parameters (haematological, somatostatin receptor positive tumour volume and general patient characteristics) between none/mild, moderate and severe haematotoxicity were identified. Persistent moderate to severe haematotoxicity was rarely observed, but after adapting dosages and/or postponed administrations, many patients still could complete their treatment course. Therefore, our selection criteria appeared appropriate for safe PRRT treatment.

## Supporting information

S1 Fig Example of Gaussian distribution. (PDF)

S2 Fig An example of liver tumour segmentation on [^68^Ga]Ga-DOTA-TATE PET/CT. (PDF)

S1 Table Haematological parameters corresponding to haematotoxicity grades in patients with adjusted therapy schedules. (PDF)

S2 Table Baseline characteristics of all patients, specified per haematotoxicity grade according to CTCAE version 5 (n (%)). (PDF)

S1 Appendix Liver tumour volume algorithm. (PDF)
