# Supplementary material for: Haematotoxicity during peptide receptor radionuclide therapy: Baseline parameters differences and effect on patient’s therapy course
Source: PLoS One. 2021 Nov 18;16(11):e0260073. doi: 10.1371/journal.pone.0260073 (PMC8601524; doi:10.1371/journal.pone.0260073)
Supplement: S1 Fig — (PDF) [file pone.0260073.s001.pdf]

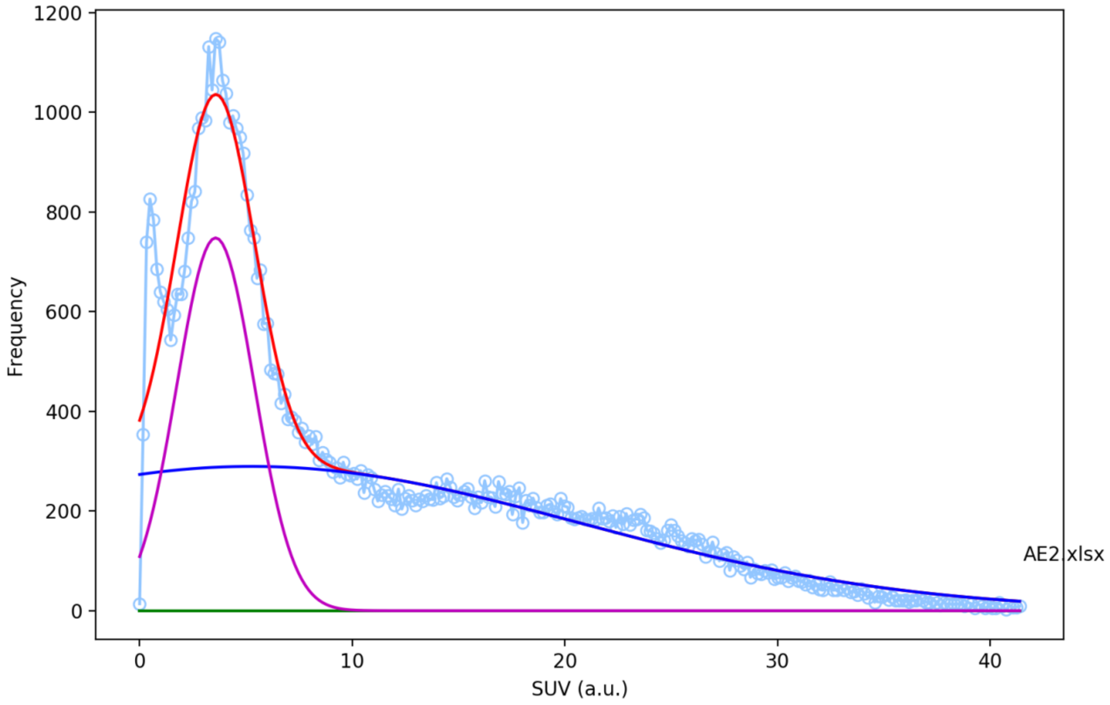

S1 Fig. Example of Gaussian distribution. The blue dots represent the binned data points and the red curve presents the sum of all fitted Gaussian functions. Normal liver tissue is represented in purple, tumour tissue in blue.
