# Supplementary figures and images for: Haematotoxicity during peptide receptor radionuclide therapy: Baseline parameters differences and effect on patient’s therapy course
Source: PLoS One. 2021 Nov 18;16(11):e0260073. doi: 10.1371/journal.pone.0260073 (PMC8601524; doi:10.1371/journal.pone.0260073)

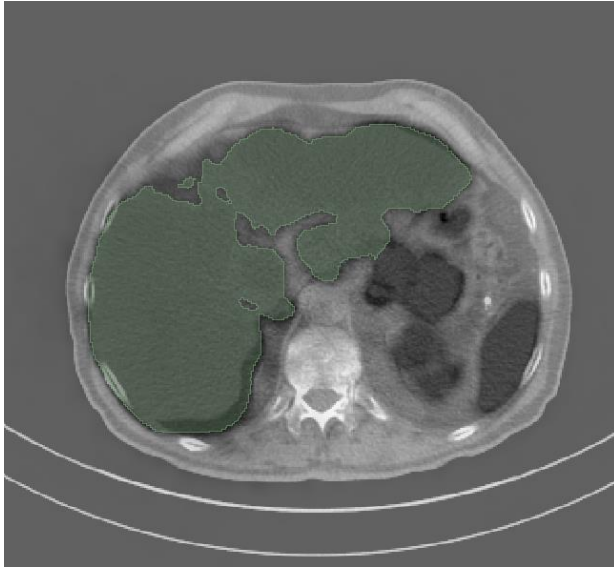

**S2 Fig. An example of liver tumour segmentation on [ $^{68}\text{Ga}$ ]Ga-DOTA-TATE PET/CT.**

Supplement: S2 Fig — (PDF) [file pone.0260073.s002.pdf]
