# Supplementary material for: Haematotoxicity during peptide receptor radionuclide therapy: Baseline parameters differences and effect on patient’s therapy course
Source: PLoS One. 2021 Nov 18;16(11):e0260073. doi: 10.1371/journal.pone.0260073 (PMC8601524; doi:10.1371/journal.pone.0260073)
Supplement: S1 Table — (PDF) [file pone.0260073.s003.pdf]

S1 Table. Haematological parameters corresponding to haematotoxicity grades in patients with adjusted therapy schedules. Grade 2 is noted as G2 and grade 3-4 as G3-4.

Pt #	After cycle 1	After cycle 2	After cycle 3	After cycle 4
1	-	G3-4: leucocytes, neutrophils, platelets	G2: leucocytes, platelets	G2: leucocytes<br>G3-4: platelets
2	G2: platelets	G2: leucocytes	G2: leucocytes, neutrophils, platelets	G2: leucocytes
3	-	-	G2: haemoglobin, leucocytes, platelets	-
4	G2: haemoglobin, neutrophils	G2: haemoglobin	G2: haemoglobin	G2: leucocytes, neutrophils, platelets<br>G3-4: haemoglobin
5	-	G2: leucocytes	G2: leucocytes	-
6	G2: platelets	-	G2: platelets	G2: platelets
7	-	G2: leucocytes	G2: leucocytes	G2: leucocytes
8	-	-	G2: haemoglobin, leucocytes	G2: haemoglobin, leucocytes
9	G2: haemoglobin	G2: haemoglobin	G2: haemoglobin<br>Discontinuation due to acute renal failure	
10	-	-	G2: haemoglobin, leucocytes, neutrophils	Discontinuation due to persistent haematotoxicity
11	G2: platelets	G2: platelets	G2: platelets	G2: haemoglobin
12	-	-	G2: leucocytes	G2: haemoglobin<br>G3-4: leucocytes, neutrophils, platelets
13	G2: leucocytes	G2: leucocytes	G2: leucocytes	G2: neutrophils<br>G3-4: leucocytes
14	-	-	G2: leucocytes, neutrophils	Discontinuation due to persistent haematotoxicity
15	G2: haemoglobin	G2: platelets<br>G3-4: haemoglobin	Discontinuation due to persistent haematotoxicity	
16	-	G2: leucocytes, neutrophils, platelets	G2: leucocytes, platelets	G2: haemoglobin, leucocytes, platelets
17	-	-	G2: haemoglobin, platelets	-
18	G2: leucocytes	G2: leucocytes, neutrophils	G2: leucocytes	G2: leucocytes, neutrophils
19	G2: leucocytes, neutrophils	G2: leucocytes	Discontinuation due to clinical deterioration	
20	G2: leucocytes		G2: leucocytes, neutrophils	G2: leucocytes
21	-	G2: platelets	G2 & G3: platelets	Discontinuation due to progressive disease
22	G2: leucocytes	G2: leucocytes	G2: leucocytes, neutrophils	Discontinuation due to persistent haematotoxicity, progressive disease and congestive heart failure
23	Ileus, treated with surgical intervention	G2: leucocytes	G2: leucocytes	G2: leucocytes
24	Fast declining platelets, therefore cycle 2 was postponed	-	-	-
