# Supplementary material for: Haematotoxicity during peptide receptor radionuclide therapy: Baseline parameters differences and effect on patient’s therapy course
Source: PLoS One. 2021 Nov 18;16(11):e0260073. doi: 10.1371/journal.pone.0260073 (PMC8601524; doi:10.1371/journal.pone.0260073)
Supplement: S2 Table — (PDF) [file pone.0260073.s004.pdf]

S2 Table. Baseline characteristics of all patients, specified per haematotoxicity grade according to CTCAE version 5 (n (%)).

**Characteristic**		**CTCAE grade**			***p* value**
		**Grade 0-1**	**Grade 2**	**Grade 3-4**	
Total number of patients (n = 100)		54 (54)	38 (38)	8 (8)	0.693
Gender	Male (n= 44)	24 (44.4)	18 (47.4)	2 (25.0)	0.509
Age at start PRRT		64.2 ± 9.0	63.5 ± 11.9	60.9 ± 14.5	
Comorbidities	Diabetes	8 (14.8)	6 (15.8)	2 (25.0)	0.763
	Cardiovascular disease	34 (63.0)	21 (55.3)	4 (50.0)	0.658
WHO performance status	WHO 0	26 (48.1)	22 (57.9)	4 (50.0)	0.454
	WHO 1	24 (44.4)	11 (28.9)	4 (50.0)	
	WHO 2/3	4 (7.4)	5 (13.2)	0	
Primary tumour site	Ileum	30 (55.6)	23 (60.5)	2 (25.0)	0.396
	Pancreas	14 (25.9)	10 (26.3)	3 (37.5)	
	Other	10 (18.5)	5 (13.2)	3 (37.5)	
Tumour grade	Grade 1	23 (42.6)	16 (42.1)	2 (25.0)	0.631
	Grade 2-3	31 (57.4)	22 (57.9)	6 (75.0)	
Functional tumour		21 (38.9)	20 (52.6)	3 (37.5)	0.395
Baseline GFR	ml/min/1.7m^2	80.4 ± 17.6	81.4 ± 23.2	86.0 ± 18.6	0.757
Baseline CgA	µg/L	820 [240-1938]	1004 [817-3093]	153 [75-2495]	0.302
Baseline bilirubin	µmol/L	8 [5-9.25]	7.5 [5-11]	8.5 [5.25-13.25	0.799
Bone metastases	Yes	29 (53.7)	19 (50.0)	3 (37.5)	0.685
	volume (ml)	10.6 [5.8-29.0]	13.0 [3.3-60.7]	20.1 [11.8-nr]	0.528
Soft tissue metastases	Yes	51 (94.4)	36 (94.7)	8 (100)	0.794
	volume (ml)	56.1 [22.4-107.8]	28.0 [18.9-88.2]	41.4 [24.7-64.4]	0.497
Liver metastases	Yes	48 (88.9)	35 (92.1)	8 (100)	0.565
	volume (ml)	699.2 [374-1243]	497.1 [284-1622]	729.7 [237-1029]	0.836
Total tumour load	volume (ml)	689.4 [390-1235]	555.1 [385-1651]	785 [279-1075]	0.986
Previous therapies	Primary tumour resection	24 (44.4)	17 (44.7)	2 (25.0)	0.563
	Loco-regional therapy	15 (27.8)	6 (15.8)	1 (12.5)	0.313
	Systemic therapy	53 (98.1)	34 (89.5)	8 (100)	0.136

Haemoglobin in mmol/L, other haematological parameters in $\times 10^9$/L. Mean ± SD and median [interquartile range]).
CgA = Chromogranin-A; GFR = glomerular filtration rate; nr = not reached.
Loco-regional treatments could include radiotherapy, radiofrequency ablation and liver (radio-) embolization, and systemic therapies could be long-acting SSA or other treatments.
