# Supplementary material for: Haematotoxicity during peptide receptor radionuclide therapy: Baseline parameters differences and effect on patient’s therapy course
Source: PLoS One. 2021 Nov 18;16(11):e0260073. doi: 10.1371/journal.pone.0260073 (PMC8601524; doi:10.1371/journal.pone.0260073)
Supplement: S1 Appendix — (PDF) [file pone.0260073.s005.pdf]

S1 Appendix. Liver tumour volume algorithm.

The low-dose CT scan acquired within the same series of the PET acquisition was resampled towards PET voxel sizes using the function “Resample Scalar/Vector/DWI Volume” in Slicer. The liver was semi-automatically segmented with “Robust Statistics Segmenter” using an estimated volume of 3500ml, intensity homogeneity of 1.0 and boundary smoothness of 0.6 and manual corrections to the segmentation were performed if deemed necessary. Accordingly, a CT mask was created and the PET voxels in this mask were extracted using MATLAB. The resulting array of voxel values were analysed using an in-house developed algorithm in Python. The 10% highest voxel values were removed to enable sufficient SUV data divided over 256 bins for a reliable fitting estimate. The histogram was fitted using a polynomial fit from which maxima and starting points were extracted to serve as input for the Gaussian fits.

Determination of liver tumour volume

Three Gaussian distribution were included in the output of the analysis. The Gaussian distribution of normal liver tissue was identified using the corresponding graphs and SUV measurements of the normal liver. Voxels were identified as tumorous voxels if the SUV was above the $\mu+\sigma$ threshold from the normal liver tissue Gaussian.
